# White Matter Dissection of the Fetal Brain

**DOI:** 10.3389/fnana.2020.584266

**Published:** 2020-09-25

**Authors:** Bianca Horgos, Miruna Mecea, Armand Boer, Bianca Szabo, Andrei Buruiana, Florin Stamatian, Carmen-Mihaela Mihu, Ioan Ştefan Florian, Sergiu Susman, Raluca Pascalau

**Affiliations:** ^1^Faculty of Medicine, Iuliu Haţieganu University of Medicine and Pharmacy, Cluj-Napoca, Romania; ^2^Department of Morphological Sciences – Anatomy and Embryology, Iuliu Haţieganu University of Medicine and Pharmacy, Cluj-Napoca, Romania; ^3^Department of Obstetrics and Gynecology, Imogen Research Center, Cluj-Napoca, Romania; ^4^Department of Morphological Sciences – Histology, Iuliu Haţieganu University of Medicine and Pharmacy, Cluj-Napoca, Romania; ^5^Department of Neurosurgery, Iuliu Haţieganu University of Medicine and Pharmacy, Cluj-Napoca, Romania; ^6^Department of Neurosurgery, Emergency County Hospital, Cluj-Napoca, Romania; ^7^Department of Pathology and Neuropathology, Imogen Research Center, Cluj-Napoca, Romania

**Keywords:** fetal brain, white matter tracts, fiber dissection, human brain development, cerebral connectivity

## Abstract

Neuroplasticity is a complex process of structural and functional reorganization of brain tissue. In the fetal period, neuroplasticity plays an important role in the emergence and development of white matter tracts. Here, we aimed to study the architecture of normal fetal brains by way of Klingler’s dissection. Ten normal brains were collected from *in utero* deceased fetuses aged between 13 and 35 gestational weeks (GW). During this period, we observed modifications in volume, shape, and sulci configuration. Our findings indicate that the major white matter tracts follow four waves of development. The first wave (13 GW) involves the corpus callosum, the fornix, the anterior commissure, and the uncinate fasciculus. In the second one (14 GW), the superior and inferior longitudinal fasciculi and the cingulum could be identified. The third wave (17 GW) concerns the internal capsule and in the fourth wave (20 GW) all the major tracts, including the inferior-occipital fasciculus, were depicted. Our results suggest an earlier development of the white matter tracts than estimated by DTI tractography studies. Correlating anatomical dissection with tractography data is of great interest for further research in the field of fetal brain mapping.

## Introduction

From ontogeny to adulthood, the brain remains the most plastic structure of the body. The formation of its cortical areas and white matter fibers is characterized by neuroplasticity. This process can be defined as the ability of the nervous system to change its activity in response to intrinsic or extrinsic stimuli by reorganizing its structure, functions, or connections. Since all the brain complexity stems from the neural tube, the fetal period corresponds to the most elaborate process of neuroplasticity, which is well regulated by genetic and molecular mechanisms.

The white matter tracts have recently come to the attention of researchers looking for a better understanding of brain functionality. The connectivity of the brain still remains a mostly uncharted area. The bundles of fibers which form the white matter can be classified in commissural (corpus callosum, anterior and posterior commissures, hippocampal commissure, and habenular commissure), projection (internal capsule), and association fibers (cingulum, superior and inferior longitudinal fasciculi, uncinate fasciculus, and inferior fronto-occipital fasciculus). Their development begins in the embryonic period and is consolidated postnatally, when myelination is still ongoing. Structural refinements in the architecture of the white matter were observed even in adolescence (Asato et al., 2010).

Recent studies have shown some of the signaling pathways involved in the development of white matter. The tracts form along the path traced by the “pioneer axons” which are guided by various molecular cues (Chédotal and Richards, 2010). This is a highly precise phenomenon, during which any disturbance can have serious consequences in postnatal life. Nonetheless, architectural reorganization and compensation may occur in pathologies like the complete or partial agenesis of the corpus callosum.

The development of fiber tracts has been studied using diffusion tensor imaging (DTI) tractography and increasingly complex imaging techniques. Used in the context of fetal MRI, tractography has the potential to diagnose anomalies in the development of the white matter tracts *in utero*. However, the major drawback of these techniques is the high number of false positive fiber tracts returned (Vasung et al., 2017), which makes its use very challenging in this particular situation, where a diagnosis can influence the decision to keep or terminate a pregnancy.

To improve the further research on the white matter, we used the classic dissection approach described by Klingler et al. (1935). This implies freezing formalin-fixed brains followed by thawing. It has been already used to describe connections in the human adult brain (Klingler and Gloor, 1960). Recently, the mechanism by which the technique allows the separation of the tracts has been studied at ultrastructural level, confirming its usefulness in separating the tracts without altering their structure (Zemmoura et al., 2016). Old anatomical techniques have paradoxically enjoyed renewed interest due to the development of newer imaging techniques (Dini et al., 2013). While no longer necessarily being viewed as a ground truth, the fiber dissection technique is nevertheless useful in the confirmation of DTI tractography and for the development of novel techniques (Dini et al., 2013; Zemmoura et al., 2014; De Benedictis et al., 2018). Although sometimes questioned, its usefulness in the field of neuroscience is confirmed by the fact that it is used in numerous recent studies (Di Carlo et al., 2019; Flores-Justa et al., 2019; Komaitis et al., 2019).

The present study is the first one to use Klingler’s dissection technique on fetal brains with the purpose of evaluating the dynamic evolution of the white matter tracts. Our approach can be further developed by association with other imagistic and molecular techniques.

## Materials and Methods

### Fetal Brains

The study was conducted on 17 normal brain hemispheres aged between 13 and 35 gestational weeks (GW). The ages of the brains were: 13, 14, 17, 20, 23, 32, 34, and 35 GW. All the brains were collected from spontaneous intrauterine death cases, caused by non-cerebral pathologies. Fetal necropsies were performed by specialized pathologists following standard protocols for intrauterine death diagnostics. The gestational age was determined based on the day of the last menstruation of the mother and on fetal ultrasonographic evaluations performed by obstetricians during the pregnancy. The brains were collected in the first 1 to 6 h after the extraction of the fetuses and they were preserved in 4% formaldehyde solution by way of intravascular perfusion and post fixation.

Some specimens included both hemispheres of the brain but some of them consisted of only one hemisphere, the other half was being utilized to histopathological examination. The study protocol was approved by the Ethics Committee of our University.

### White Matter Dissection

For the tridimensional white matter dissection, the brains were prepared according to the Klingler method (Ludwig and Klingler, 1956; Pascalau et al., 2016). They were immersed in plastic recipients filled with 10% formaldehyde solution and frozen at −20°C for 14 days. Afterward, they were thawed gradually at room temperature or under running warm water for several hours. As a result, the gray matter structures became friable and the white matter fibers where easy to separate from one another. The fiber dissection technique implies exposing the most accessible portions of the white matter tracts by cortex removal, following the fibers to their terminations and extracting each tract to reveal the one underneath. In adult human brains and in some mammalian brains, it is usually performed with blunt wooden instruments made from tongue depressors (Türe et al., 2000; Pascalau and Szabo, 2017). However, these instruments where too gross for the fetal brains, so Wheeler spatulas were used instead. Serial photographs were taken at each dissection step, maintaining the same distance and angle relative to the specimens.

Since no cerebral pathology was expected based on *in utero* imaging investigations, the latero-medial and medio-lateral stepwise fiber dissection protocols used previously by our group in the adult human brain (Pascalau et al., 2018) and in some other mammalian brains (Ludwig and Klingler, 1956; Türe et al., 2000; Pascalau et al., 2018) were employed, with some changes. The steps of the two protocols where intermingled (Table 1 and Supplementary Video 1) in order for them to be performed on the same hemisphere without making it too fragile. As the existence and development level of the tracts could not be anticipated, the usual landmarks could not be used, so the dissection had to be performed blindly (gray matter and short association fibers where carefully removed until some parallel fibers where encountered, and the latter where followed to their terminations, which gave away the identity of the tracts).

**TABLE 1:** Stepwise dissection protocol.

**Step**	**Procedure**	**Exposed white matter tracts**
1	Sagittal section along the midline	n/a
2	Cortex removal from the medial surface	Cingulum
3	Cingulum removal	Corpus callosum
4	Hemisphere flipped on the lateral surface	n/a
5	Cortex removal from the lateral surface	Short association fibers
6	Exposure of long fibers around the insular cortex	Uncinate fasciculus Superior longitudinal fasciculus
7	Exposure of long fibers in the latero-basal area	Inferior longitudinal fasciculus
8	Removal of the three previous fascicles and of the white matter/gray matter layers underlying the insular cortex	Fronto-occipital fasciculus
9	Removal of the fronto-occipital fasciculus	Internal capsule (lateral aspect)
10	Hemisphere flipped on the medial surface and removal of the 3rd ventricle wall	Pillars of fornix trunk of anterior commissure
10’	Removal of short fibers in the orbitofrontal region	Anterior projections of anterior commissure
10”	Removal of posterior projections of anterior commissure	Optic radiations
11	Removal of body of corpus callosum	Body of fornix
12	Extraction of fornix, caudate nucleus, and thalamus	Internal capsule (medial aspect)

The protocol began with the separation of the brain hemispheres with a sagittal section along the midline (Step 1). The cortex on the medial surface of the hemisphere was removed first and the fibers of the cingulum where exposed along their trajectory (Step 2). Then, the cingulum was removed and the underlying corpus callosum was dissected (Step 3). At this stage, the hemisphere was switched to its lateral surface (Step 4) and the cortex on this surface was removed (Step 5). The long association fascicles that were present at the particular gestational age were exposed and removed one by one in a latero-medial order. Depending on the degree of development, the insular cortex or the sylvian fissure were used as principal landmarks for the dissection of the most superficial tracts, namely the uncinate fasciculus on the ventral part and the superior longitudinal fasciculus on the dorsal side of this region (Step 6). The inferior longitudinal fasciculus was identified as being located between the previous tracts (Step 7). The protocol continued with the removal of the layer formed by these fascicles and with the dissection of the successive white matter/gray matter layers underlying the insular cortex (extreme capsule, claustrum, external capsule, and lenticular nucleus) resulting in the exposure of the fronto-occipital fasciculus (Step 8). By removing this fasciculus, the lateral surface of the internal capsule was reached (Step 9). At this point, the hemisphere was switched again on its medial side and the dissection of the pillars of fornix situated under the lateral wall of the third ventricle was performed (Step 10). In the situations where it was not too small, the trunk of the anterior commissure was identified immediately anterior to the pillars of fornix and its anterior projections were followed in the orbitofrontal region (Step 10’). Its posterior projections were followed as they ran on the lateral surface and they were removed in order to completely uncover the optic radiations (Step 10”). Next, the body of corpus callosum was lifted and the frontal horn of the lateral ventricle was opened. The head of the caudate nucleus and the body of fornix were visible at this stage of the dissection (Step 11). The columns of fornix where followed in the temporal horn of the ventricle, which was also opened, and the fornix was extracted together with the hippocampus uncovering the dorsal surface of the thalamus and the body and tail of the caudate nucleus, which were removed in the final step of the dissection in order to expose the medial surface of the internal capsule (Step 12).

## Results

### Developmental Changes in the External Configuration

**FIGURE 1:**
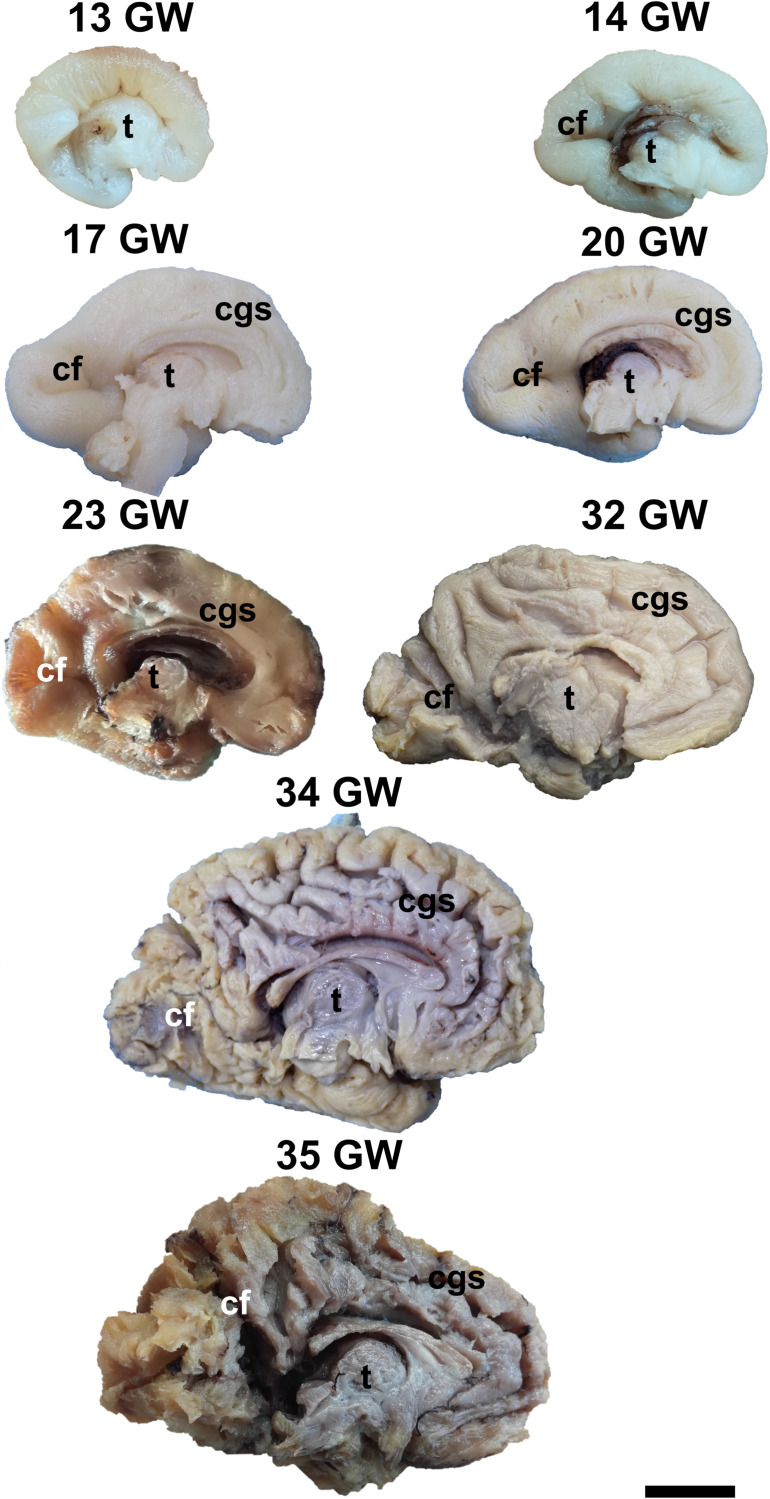
Medial surface of the cerebral hemispheres. cf, calcarine fissure; cgs, cingulate sulcus; t, thalamus. Scale bar = 5 cm.

**FIGURE 2:**
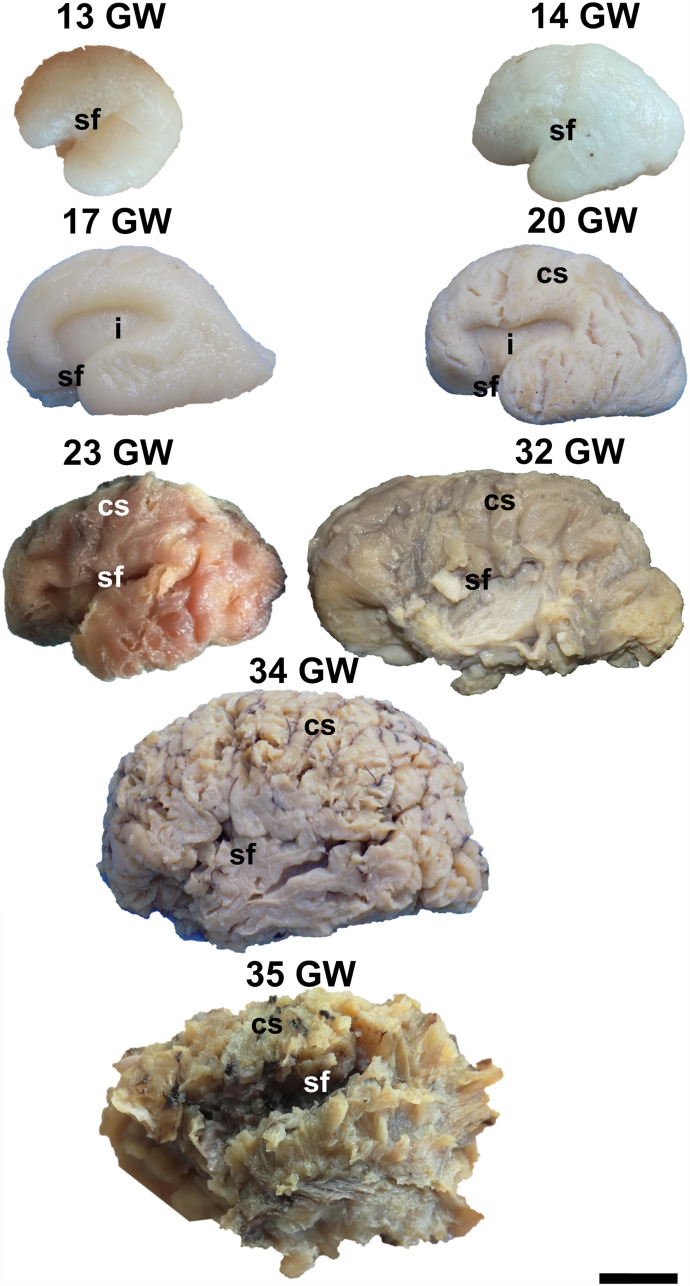
Lateral surface of the cerebral hemispheres. cs, central sulcus; i, insula; sf, sylvian fissure. Scale bar = 5 cm.

During the interval of development that was investigated here, 13–35 weeks of gestation, the volume of the brain increases almost three times (from a longitudinal diameter of 3 cm at 13 weeks to one of 8.5 cm at 35 weeks). The shape of the brain evolves from a globular one (vertical diameter: longitudinal diameter ≈4:5) to an ovoid (vertical diameter: longitudinal diameter ≈3:5). The aspect of the cortex is flat between 13 and 17 weeks of gestation. The primary sulci appear at 20 weeks and the secondary and tertiary sulci are observed by the gestational age of 34 weeks (Figures 1, 2).

On the medial surface of the hemisphere (Figure 1), the calcarine fissure can already be seen at 13 weeks. It is wide and linear and it becomes narrower and takes its characteristic “Y” shape by 17 weeks. The cingulate sulcus appears on the frontal side at 17 weeks and then progresses posteriorly. On the lateral surface of the brain (Figure 2), the anterior part of the sylvian fissure appears as a shallow sulcus at 13 weeks. Then, at 14 weeks, it bifurcates and forms the periinsular sulcus. At 20 weeks of gestation, the frontal operculum begins to grow and to cover the insula dorsally, followed by the temporal operculum which covers the ventral part of the insula by 23 weeks of gestation, so that the sylvian fissure is at this time a deep sulcus. The central sulcus firstly appears at 17 weeks. Its position moves posteriorly in the later stages due to the faster growth of the frontal lobe.

### Development of the White Matter Tracts in Normal Fetal Brains

For a white matter tract to be identified at a certain age by fiber dissection, the following conditions had to be fulfilled: (I) the tract should have been in the appropriate position relative to the surface of the brain; (II) a consistent bundle of fibers with parallel directions could be followed for at least one centimeter on the predicted trajectory of the tract and (III) the consistency of the fibers, subjectively assessed by the dissector based on the resistance they opposed to the dissection instrument, should have been greater than that of the tissues surrounding it. Based on these criteria nine fiber tracts have been identified, which cover the major fasciculi in the human brain.

According to the gestational ages at which they could be identified, there are four waves of emerging white matter tracts (Table 2). The first wave consists of tracts that were already present (identifiable, not necessarily fully formed) at the earliest gestational age included in this investigation, namely, 13 GW. It comprises the commissural tracts (corpus callosum, fornix and the anterior commissure), and an association tract, the uncinate fasciculus. The second wave was identified at 14 GW and consists of three association tracts: the superior longitudinal fasciculus, the inferior longitudinal fasciculus, and the cingulum. The projection fibers, known as the internal capsule, account for the third wave of development, occurring at 17 GW. The fourth wave of development appeared at 20 GW and is represented by the appearance of the inferior fronto-occipital fasciculus.

**TABLE 2:** Developmental waves in the formation of the white matter tracts.

**Developmental wave**	**Gestational age (weeks)**	**White matter tracts**
I	13	Corpus callosum Fornix Anterior commissure The uncinate fasciculus
II	14	Superior longitudinal fasciculus Inferior longitudinal fasciculus Cingulum
III	17	Internal capsule
IV	20	Inferior fronto-occipital fasciculus

### The Corpus Callosum

The corpus callosum is a commissural tract located in the superior half of the hemispheres. It connects the following areas on the two hemispheres: frontal pole, prefrontal area, premotor area, motor area, somatosensory area, superior parietal lobe, inferior parietal lobe, occipital lobe and posterior temporal lobe.

**FIGURE 3:**
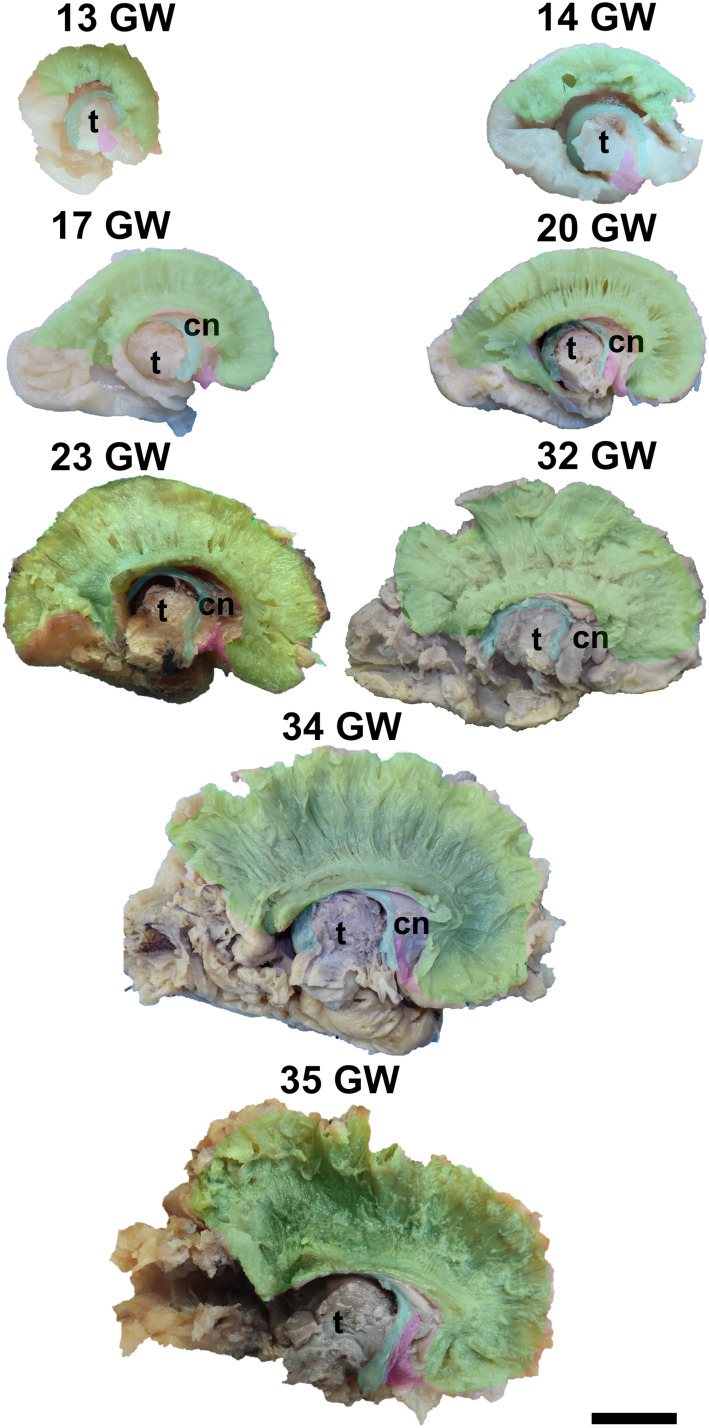
Early tracts dissected from the medial surface of the hemisphere. green, corpus callosum; cyan, fornix; magenta, anterior commissure; cn, caudate nucleus; t, thalamus. Scale bar = 5 cm.

It is already recognizable on the medial surface of the hemispheres (Figure 3). Its dorsal surface can be exposed completely by removing the cortex and the short association fibers from the medial aspects of the frontal, parietal and occipital lobes, and after extracting the cingulum (Supplementary Video 1). Although it is identifiable at 13 GW, its fibers are not fully developed yet. At this age, the consistency and the individualization of the fibers is better in a sector located in the frontal part of the brain. With the increase in gestational age, this sector expands in an antero-posterior direction until the age of 23 GW, when the callosal fibers start to look uniform. The fraction of the brain volume occupied by the corpus callosum also increases with age. While at 13 weeks it occupies around 1/2 of the fronto-occipital diameter of the hemisphere brain, at 35 GW it makes up for more than 2/3 of the same diameter brain.

### The Fornix

The fornix is a mostly commissural tract that also contains association fibers. It encircles the thalamus, has close relations with the cerebral ventricles and the caudate nucleus, and connects the hippocampus, amygdalae, mammillary bodies, and the accumbens nuclei.

The complex tridimensional architecture of the fornix can be revealed *in situ* only partially with our protocol because of the tract’s deep position in the lateral ventricle. The pillars of fornix and the anterior part of its body can be visualized by removing the ependymal layer of the third ventricle (Figure 3 and Supplementary Video 1). The posterior part of the body, the crura and fimbria can only be seen if the fornix is pulled out from the ventricles. Its appearance is already established at 13 GW and does not change significantly in the following stages of development except for the fact that its volume grows slowly than that of other structures of the brain so that decreases relative to the total volume of the brain. A possible explanation could be the fact that the fornix connects the archaic structures of the brain, so it is surpassed in growth rate by the tracts that connect neopallial areas.

### The Anterior Commissure

The anterior commissure consists of a trunk located within the anterior wall of the third ventricle, lamina terminalis, and two sets of projections connecting the amygdalae and the anterior temporal lobes with the fronto-orbital areas.

The posterior projections of the anterior commissure are identifiable from their lateral surface at 13 GW, being located above the uncinate fasciculus (Figure 3). As the development advances, their location becomes deeper, as they are covered by the later emerged tracts of the ventral fronto-temporal area. The trunk and anterior projections are apparent only later, namely at 17 weeks of gestation. While at early ages they are almost at the basal surface of the brain, later they become more profound, and the cortex and the short association fibers of the subgenual area and the cingulum need to be removed in order for them to be revealed (Supplementary Video 1).

### The Uncinate Fasciculus

The uncinate fasciculus is the earliest identified association tract. It is shaped like a hook and resides in the floor of the sylvian fissure, making connections between the orbitofrontal cortex and the temporal pole.

**FIGURE 4:**
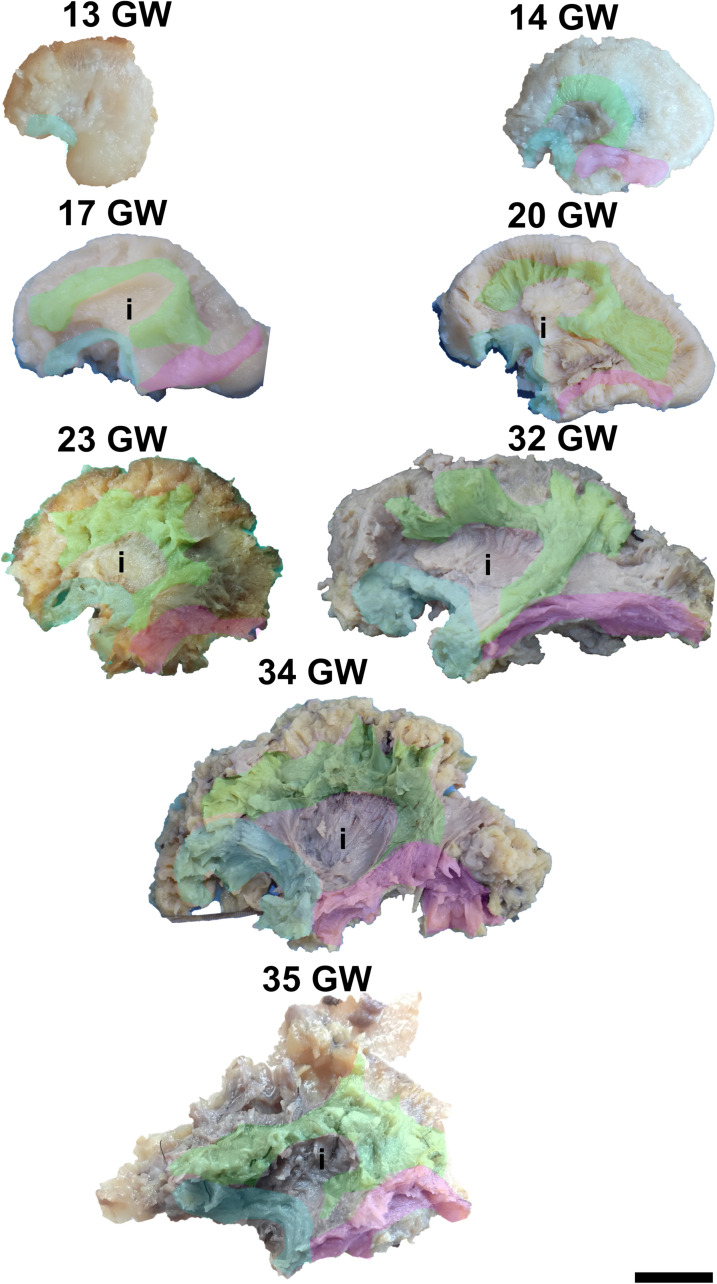
Early tracts dissected from the lateral surface of the hemisphere. green, superior longitudinal fasciculus; cyan, uncinate fasciculus; magenta, inferior longitudinal fasciculus; i, insula. Scale bar = 5 cm.

Its superficial position allows for its exposure just by removing the cortex of the region as early as at 13 weeks of gestation (Figure 4). Its frontal projections are initially better developed than the temporal ones, which catch up by 23 weeks of gestation. The initial aspect of the tract is that of a thin bundle of parallel fascicles which gets thicker and gains more spread terminations as the developmental process advances.

### The Superior Longitudinal Fasciculus

The superior longitudinal fasciculus has a superficial latero-dorsal position in the cerebral hemisphere. It is an multi-component association tract composed of a deep, direct component (the arcuate fasciculus), connecting the inferior frontal gyrus (Broca area) to the posterior and middle thirds of the superior temporal gyrus (Wernicke area – Word Form Area), middle temporal gyrus, and the middle third of the inferior temporal gyrus and a more superficial indirect component, the anterior and the posterior tracts, connecting the inferior frontal gyrus with the inferior parietal lobule (Geschwind area) and the inferior parietal lobule with the posterior portion of the superior temporal gyrus, respectively.

It can be dissected by removing the periinsular cortex in the early stages, or the frontal and temporal opercula in the later stages of development (Figure 4). By 14 weeks of gestation, the immediately periinsular portion of the tract is identified by dissection. The fibers follow mostly parallel directions. These early fibers most probably correspond to the arcuate fasciculus. They terminate in restricted areas in the inferior frontal and superior temporal regions. The frontal and temporal terminations begin to spread at 17 weeks. At 20 weeks, terminations in the parietal cortex start to be seen and the tract becomes wider, as a sign that the anterior and posterior tracts of the indirect component are developing at this time. Starting with the gestational age of 23 weeks, the tract gains more and more radiating fibers and it becomes multilayered. The newer fibers are added always in more superficial positions compared to the older ones. The tract also occupies larger and larger fractions of the brain’s volume.

### The Inferior Longitudinal Fasciculus

The inferior longitudinal fasciculus is an association tract which occupies a baso-lateral position in the brain, running from the temporal pole to the visual association areas in the occipital pole. It is the most superficial tract of the temporo-occipital region.

For the dissection of the inferior longitudinal tract, the cortex and the short association fibers of the inferior portions of the temporal and occipital lobes have to be removed. The tract can be identified starting with 14 weeks of gestation (Figure 4). With the advancement in gestational age, its length increases and its shape evolves from a slim band of parallel fibers to a more tridimensional structure, with diverging fibers that join it and leave it for partial lengths along its trajectory.

### The Cingulum

The cingulum is the only major association tract located on the medial region of the hemisphere. It encircles the corpus callosum and the hippocampus and connects the cingulate gyrus, the parahippocampal gyrus, the uncus, the amygdala, and the medial surfaces of the frontal and parietal lobes.

**FIGURE 5:**
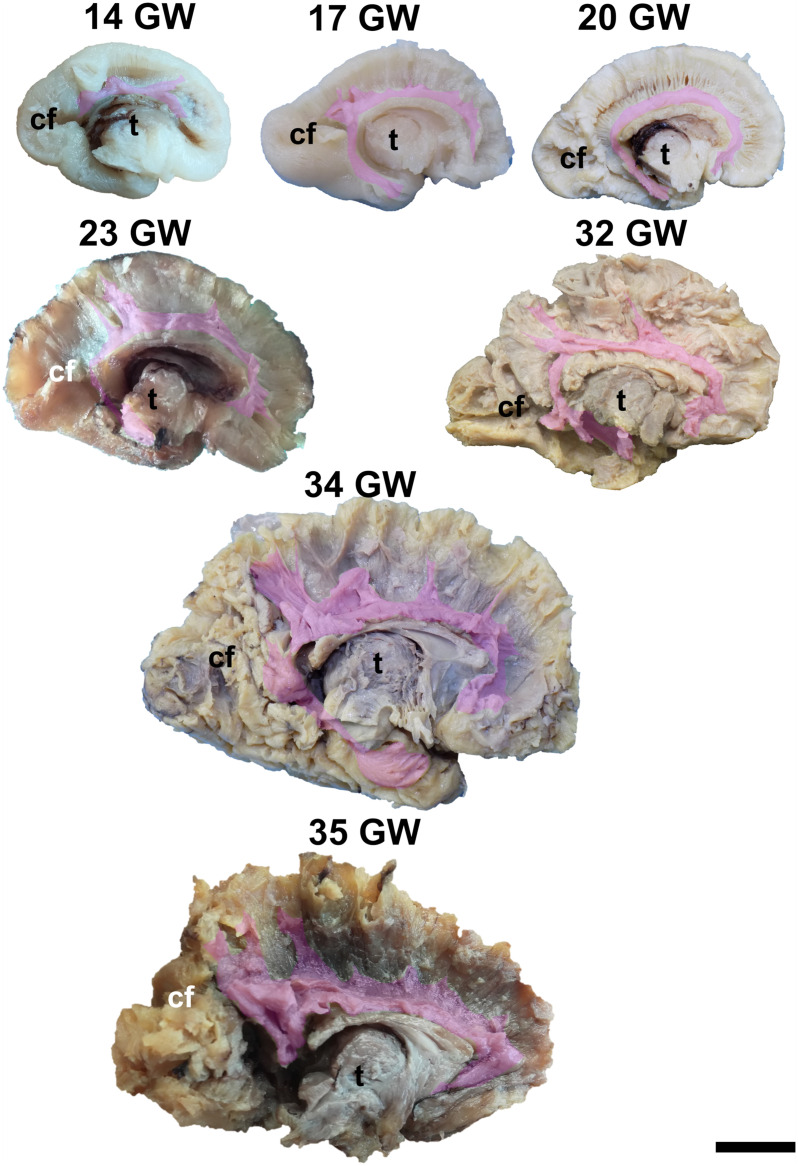
Cingulum. cf, calcarine fissure; t, thalamus. Scale bar = 5 cm.

The dissection of the cingulum involves the removal of the cortex of the medial and basal surfaces of the cerebral hemispheres. It can be identified as early as at 14 weeks of gestation (Figure 5). Its terminations in the subgenual area and at the uncus are well defined by 17 weeks. Later on, radiating fibers leave its trajectory and spread in the entire medial cortex.

### The Internal Capsule

The internal capsule is a region in the center of each cerebral hemisphere where all the projection fibers converge in their path from/to the cortex, thalamus, basal ganglia, brainstem nuclei and spinal cord. Its fibers are in close vicinity to the basal ganglia, the thalamus and the association tracts, and are intersected perpendicularly by the callosal fibers.

**FIGURE 6:**
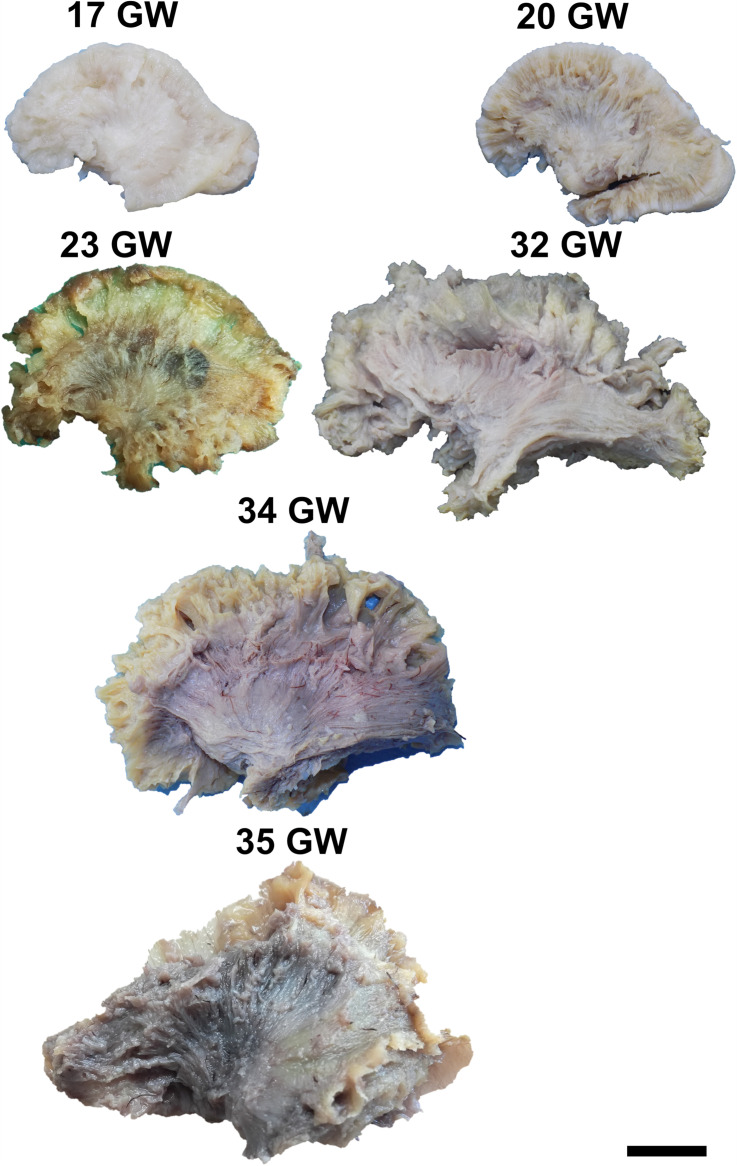
Lateral aspect of the internal capsule. Scale bar = 5 cm.

To expose this tract, either from the medial surface or from the lateral surface of the hemispheres, one needs to remove all the other gray matter and white matter structures of the brain. The tract meets all the identifying criteria at 17 weeks of gestation although vertical fibers can already be seen at 13 weeks of gestation. These are considered to belong to the radial glia (Figure 6). The length of the fibers increases with the gestational age.

### The Inferior Fronto-Occipital Fasciculus

The inferior fronto-occipital fasciculus is the deepest located association tract. It connects the inferior frontal gyrus, the middle frontal gyrus, the frontal pole, the orbitofrontal gyri, the superior parietal lobule, the occipital pole, and the visual association areas.

**FIGURE 7:**
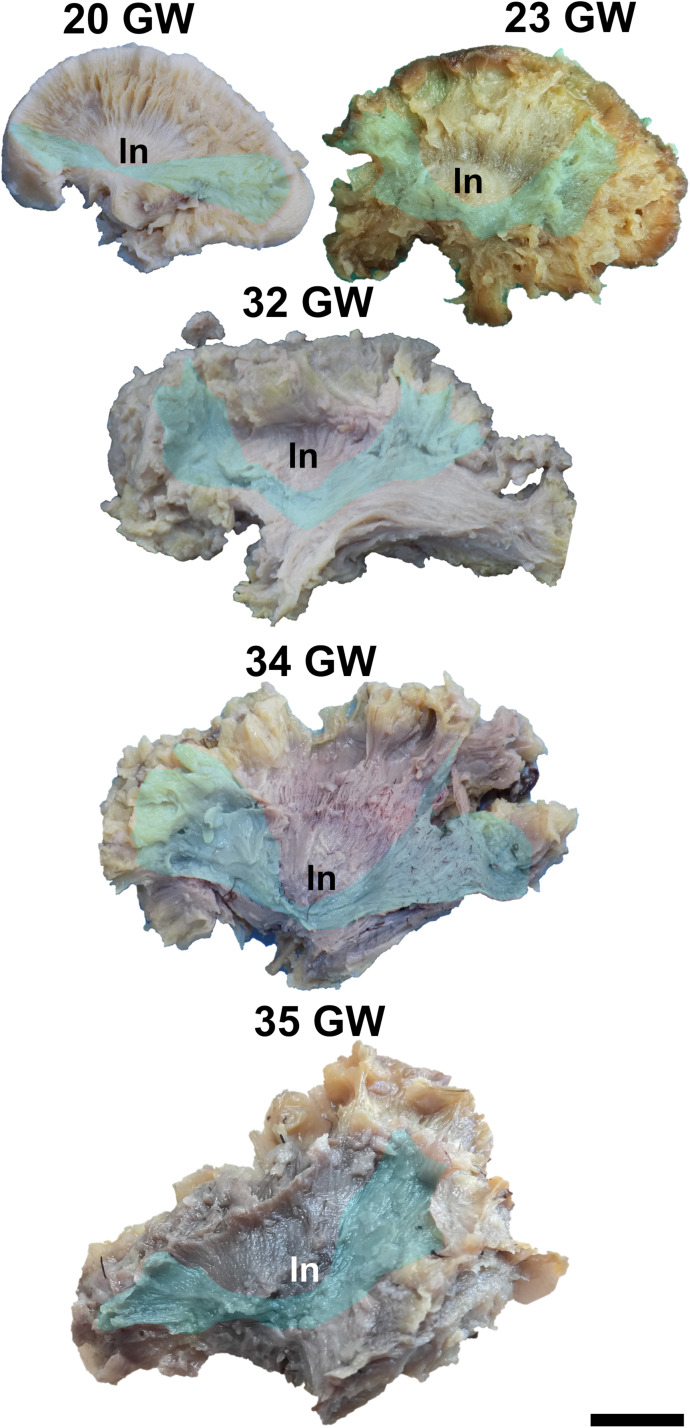
Inferior fronto-occipital fasciculus. ln, lenticular nucleus. Scale bar = 5 cm.

It can be exposed after the extraction of the cortex of the lateral surface of the brain, followed by the short association tracts, the superior longitudinal fasciculus, the insular cortex and the claustrum. The fiber dissection technique allows the identification of this tract starting with 20 weeks of gestation (Figure 7). In the next stages the fibers lengthen, and new fibers are added, so the tract becomes wider.

## Discussion

In the present study, we have investigated the trajectory and architecture of the cerebral white matter by means of gross dissection.

Our findings indicate that the major white matter bundles emerge in four waves of development. The first wave consists of tracts already present in our youngest specimens, namely at 13 GW, and includes: the corpus callosum, the fornix, the anterior commissure and the uncinate fasciculus. What is gained by employing this technique is an even earlier observation of the fascicles compared to what can be accomplished through traditional tractography. While the fornix and the anterior commissure can be observed by DTI in 13 weeks specimens (Ren et al., 2006; Huang et al., 2009), the uncinate fasciculus and the corpus callosum become visible as late as 15 weeks (Huang et al., 2009). Since DTI measures water diffusion inside myelinated axon fibers and does not directly visualize the fibers, anatomical dissection and histology reports add valuable information. Consistent with our results, immunohistochemistry studies showed that the pioneering axons of the corpus callosum appear at around 13–14 weeks (Rakic and Yakovlev, 1968; Ren et al., 2006). However, hematoxylin and eosin staining revealed that the anterior commissure appears even earlier (12 weeks) than the corpus callosum (Cho et al., 2013). In mice, carbocyanine dyes injected into the cingulate cortex or into the neocortex revealed that the cingulate axons are the first to cross the midline in order to form the corpus callosum (Rash and Richards, 2001). The cingulate axons are also projected toward the fornix to the ipsilateral hippocampus. Thus, pioneering axons from the cingulate cortex contribute, at least partially, to the first wave of development.

The second wave involves fibers observed at 14 GW, such as the superior and inferior longitudinal fasciculi, as well as the cingulum. Again, our results suggest that these tracts appear earlier than estimated by DTI. Data from the literature indicates that the inferior longitudinal fasciculus can be identified by DTI at 19 weeks, the cingulum can be traced at 17–19 weeks and the superior longitudinal fasciculus cannot be appreciated even at birth (Zhang et al., 2007). Although the projections of the cingulum are very complex, the pioneering cingulate axons could also be responsible for this wave of development. Agenesis of corpus callosum is often associated with smaller dimensions of the cingulum (Nakata et al., 2009), linking the cingulate cortex in their development. Interestingly, cortical-cortical connections (superior and inferior longitudinal fasciculi) were speculated to appear later (Dubois et al., 2015), but histology studies regarding neocortical pioneering axons are lacking.

The internal capsule fulfilled our tract identification criteria at 17 weeks of gestation, accounting for the third wave. Although some vertical fibers were identified using Klingler’s dissection at 13 weeks, they did not meet two of our three criteria (i.e., traceable course for at least 1 cm and fiber consistency), so we considered them to belong to the radial glia, which is known to have the same position (Dubois et al., 2015). DTI studies suggested the same development of the internal capsule starting from 13 weeks (Huang et al., 2009; Dubois et al., 2015).

Finally, all major tracts, including the inferior fronto-occipital fasciculus, can be well identified at 20 GW, thus completing the fourth wave of development. DTI studies can trace the inferior fronto-occipital fasciculus in 19 weeks brains, along with the inferior longitudinal fasciculus and the cingulum (Huang et al., 2009). However, the lack of 18–19-week specimens in our study is an impediment to precisely dating the emergence of this tract, and it may appear earlier.

The age differences in our study compared to previous ones using different methods show that the order of development of the white mater tracts is still controversial. The fact that gestational age is always estimative and that the criteria for its approximation may differ from one study to another can play a role in these discrepancies. Moreover, Klingler’s dissection consists of subjective observations made by the dissector and cannot always follow a strict protocol (Türe et al., 2000). Another major limitation of this technique is that it requires postmortem specimens, and inadequate fixation and preservation can hinder the accuracy of the dissection.

From a clinical point of view, the developmental waves described in this study can be linked with possible neurocognitive implications of lesions occurring at particular gestational ages based on the current knowledge on the cognitive roles played by different tracts in the adult. Starting with the first wave of development (13 weeks), we can notice that it lays the foundation of social interaction and memory. Thereby the corpus callosums functions are: interhemispheric transfer of information, integration of inputs from one or both hemispheres, facilitation of some cortical activities, and inhibition of cortical functions (Lavrador et al., 2019). The fornix is involved in spatial processing and episodic memory (Dumont et al., 2015; Hodgetts et al., 2015; Senova et al., 2020), as well as pattern separation (Bennett et al., 2015). There is also emerging evidence of the dorsal hippocampal commissure contributing to recognition memory (Postans et al., 2020). The anterior commissure’s functional implications in humans are still somewhat elusive, appearing to be related to olfactory and non-visual interhemispheric communication (Gerrish et al., 2014). Studies on mice have found that the transection of this fascicle results in higher motor activity, aberrant social interaction and reduced associative memory, behavioral features associated with neuropsychiatric disorders, such as autism spectrum disorders (Hsu et al., 2020). The uncinate fasciculus is reported to contribute to reward/punishment based learning (Von Der Heide et al., 2013), facial emotional processing (Coad et al., 2017), emotional empathy (Oishi et al., 2015), and self-regulation (Passamonti et al., 2012). It is also associated with higher level object perception and object memory (Murray et al., 2005). Although the exact role in language is debated (Duffau et al., 2009; Harvey et al., 2013), there is evidence to support its involvement in naming (Papagno, 2011; Papagno et al., 2011; Zhang et al., 2018).

The second and fourth waves are mostly involved in language formation. The tracts involved can be divided into two streams: the dorsal stream, involving the arcuate fasciculus, which processes the phonological aspects of language (Almairac et al., 2015), and the ventral stream, containing the inferior fronto-occipital fasciculus, with a role in the semantic processing of language (Duffau, 2008; Herbet et al., 2017). We can observe that the semantic aspects of language develop later (in the fourth wave of development – 20 weeks) than the phonological ones (second wave – 14 weeks). The inferior fronto-occipital fasciculus also appears to be involved in reading and writing (Catani and Mesulam, 2008; Motomura et al., 2014; Rollans and Cummine, 2018), and “on line” motor planning subserving the dorsal visual stream (Rizzolatti and Matelli, 2003). Furthermore the superior longitudinal fasciculus has been shown to play a role in working memory performance (Bathelt et al., 2019) and in intelligence (Simpson-Kent et al., 2020). The inferior longitudinal fasciculus is proposed to be part of the ventral visual pathways and appears to have a “multi-function” role. It is involved in object identification (Fernández Coello et al., 2013; Herbet et al., 2018), face recognition and processing (Herbet et al., 2018; Hodgetts et al., 2015), picture naming (Herbet et al., 2019), semantic autobiographical memory, visual memory, visual emotion, and reading (Herbet et al., 2018).

Regarding the cingulum, it has been demonstrated that a higher microstructural organization of this fascicle is linked with a higher cognitive performance (Bathelt et al., 2019). It is involved in spatial and working memory, emotion, pain, motivation, error detection, and response selection (Bubb et al., 2018).

The third wave is responsible for the development of the internal capsule, which provides sensorimotor connections (Catani and Mesulam, 2008).

## Conclusion

The objective of the study was to establish the brain architecture at different gestational ages, opening a path for further research on the fetal brain architecture in more complex brain abnormalities. Neuroplasticity can determine fibers rearrangement in different neuro-developmental pathologies (e.g., Probst bundles in corpus callosum agenesis), leading to normal functioning brains (Nakata et al., 2009). Having a template of the normal architecture of the white matter tracts in a developmental timeline is essential for the future use of tractography as an evaluation tool for cerebral development, especially as it may be an important factor in the abortion decision process.

## Data Availability Statement

All datasets presented in this study are included in the article/Supplementary Material.

## Ethics Statement

The studies involving human participants were reviewed and approved by the Ethics Committee of Iuliu Haţieganu University of Medicine and Pharmacy, Cluj-Napoca. The Ethics Committee waived the requirement of written informed consent for participation.

## Author Contributions

RP, SS, and BS: study design. SS and FS: brain collection. BH, MM, ABo, and RP: dissections. RP: image processing. BH, RP, MM, ABo, ABu, and SS: manuscript preparation. SS, RP, BH, MM, ABo, ABu, BS, IS, and C-MM: manuscript review. All authors contributed to the article and approved the submitted version.

## Conflict of Interest

The authors declare that the research was conducted in the absence of any commercial or financial relationships that could be construed as a potential conflict of interest.

## Supplementary Material

Supplementary Video 1 Stepwise fiber dissection protocol for the fetal brain. Unprocessed successive images from a stepwise fiber dissection performed on the left hemisphere of a 34 gestational weeks old fetal brain.
